# KCl -Permeabilized Pancreatic Islets: An Experimental Model to Explore the Messenger Role of ATP in the Mechanism of Insulin Secretion

**DOI:** 10.1371/journal.pone.0140096

**Published:** 2015-10-07

**Authors:** Javier Pizarro-Delgado, Jude T. Deeney, Rafael Martín-del-Río, Barbara E. Corkey, Jorge Tamarit-Rodriguez

**Affiliations:** 1 biochemistry Department, Medical School, Complutense University, Madrid, Spain; 2 Research Department, “Ramón y Cajal” Hospital-IRYCIS, Madrid, Spain; 3 Obesity Research Center, Department of Medicine, Boston University School of Medicine, Boston, Massachusetts, United States of America; Broad Institute of Harvard and MIT, UNITED STATES

## Abstract

Our previous work has demonstrated that islet depolarization with KCl opens connexin36 hemichannels in β-cells of mouse pancreatic islets allowing the exchange of small metabolites with the extracellular medium. In this study, the opening of these hemichannels has been further characterized in rat islets and INS–1 cells. Taking advantage of hemicannels’opening, the uptake of extracellular ATP and its effect on insulin release were investigated. 70 mM KCl stimulated light emission by luciferin in dispersed rat islets cells transduced with the fire-fly luciferase gene: it was suppressed by 20 mM glucose and 50 μM mefloquine, a specific connexin36 inhibitor. Extracellular ATP was taken up or released by islets depolarized with 70 mM KCl at 5 mM glucose, depending on the external ATP concentration. 1 mM ATP restored the loss of ATP induced by the depolarization itself. ATP concentrations above 5 mM increased islet ATP content and the ATP/ADP ratio. No ATP uptake occurred in non-depolarized or KCl-depolarized islets simultaneously incubated with 50 μM mefloquine or 20 mM glucose. Extracellular ATP potentiated the secretory response induced by 70 mM KCl at 5 mM glucose in perifused rat islets: 5 mM ATP triggered a second phase of insulin release after the initial peak triggered by KCl-depolarization itself; at 10 mM, it increased both the initial, KCl-dependent, peak and stimulated a greater second phase of secretion than at 5 mM. These stimulatory effects of extracellular ATP were almost completely suppressed by 50 μM mefloquine. The magnitude of the second phase of insulin release due to 5 mM extracellular ATP was decreased by addition of 5 mM ADP (extracellular ATP/ADP ratio = 1). ATP acts independently of K_ATP_ channels closure and its intracellular concentration and its ATP/ADP ratio seems to regulate the magnitude of both the first (triggering) and second (amplifying) phases of glucose-induced insulin secretion.

## Introduction

Rat islets stimulated with 10 mM α-ketoisocaproic acid (KIC) respond with a biphasic secretion of insulin of smaller magnitude than that triggered by 20 mM glucose (1). Paradoxically, the simultaneous depolarization with 70 mM KCl almost completely suppressed KIC-induced second phase of release. Failure to stimulate a second phase of secretion correlated with an increased release of GABA and a corresponding decrease of the islet amine content [1]. Glucose-induced insulin secretion was less affected by the simultaneous depolarization with 70 mM KCl [2]. A careful study of islet cells’ permeability to adenine nucleotides revealed that a gradual depolarization with KCl (15 to 70 mM) at 5 mM glucose induced a parallel decrease of ATP content that could be reversed by increasing the extracellular ATP concentration in the milimolar range [3]. This increase of β-cell plasma membrane permeability was attributed to the opening of connexin 36 (*Cx36*) hemichannels: it was blocked by either deletion of *Cx36* (mouse germ cell knockout), glucose in the range 5 to 20 mM, or pharmacological inhibition with mefloquine in both pancreatic mouse islets and *Xenopus Laevis* oocytes overexpressing *Cx36* [3].

*Cx36* has been identified as the principal molecular component of gap junction channels between β-cells, both in rodents and humans [4]. These intercellular channels provide the needed synchronization of membrane depolarization, spike activity and cytosolic calcium oscillations among β-cells for an appropriate glucose-induced insulin release [5]. Many connexin isoforms are also able to form open hemichannels for rapid exchange of ions, second messengers and metabolites between the cell interior and interstitial space with an exclusion limit close to 1KD [6, 7, 8].

In this paper we have further investigated the responsible mechanism of the increased plasma membrane permeability induced by KCl depolarization in rat islets. Moreover, we have devised an artificial system, “an islet permeabilized model” that allows evaluation of the effects of ATP, independent of the KATP channel, on insulin secretion. It has been found that depolarized islets are permeable to extracellular ATP which increases the intracellular nucleotide concentration as well as the ATP/ADP ratio and stimulates a second phase of insulin secretion after the first one triggered by KCl depolarization itself.

## Materials and Methods

### Materials

Collagenase P and FA-free bovine serum albumin were obtained from Roche Diagnostics S.L. (Barcelona, Spain). Bovine serum albumin and most of the substances, inhibitors (POM–1, NPPB, carbenoxolone, flufenamic acid, mefloquine, diazoxide), activators (bzATP), enzymes and coenzymes were obtained from Sigma-Aldrich Química S.A. (Madrid, Spain). Other inhibitors used (ARL–67156, CGS15943, suramin) were from Tocris Bioscience (Biogen Científica S.L., Spain). Rat insulin standards were from Linco Research, Inc. (St. Charles, Missouri, U.S.A.). Na^125^I was obtained from PerkinElmer España, S.L. (Madrid, Spain). Ad-CMV-Luciferase was from Vector Biolabs (Philadelphia, PA, U.S.A.). Inorganic compounds and organic solvents were obtained from VWR International Eurolab S.L. (Spain).

### Cellular transduction assays

INS–1 832/13 cells were cultured in RPMI 1640 medium supplemented with 10 mM HEPES, 1 mM pyruvate, 50 μg/ml streptomycin, 50 IU/ml penicillin, 50 μM ß-mercaptoethanol and 10% FBS (Life Technologies, NY). Animal care, use and experimental protocols were submitted and approved by the Ethics Committee of Complutense University, responsible for the correct application of the order 86/ 609 / CEE (Spanish order 1201/2005). Islets were isolated from the pancreas of male Wistar-albino rats (250–275 g BW) by collagenase digestion and cultured in RPMI 1640 supplemented with 50 μg/ml streptomycin, 50 IU/ml penicillin and 10% FBS. After overnight culture islets were dissociated into single cells by gentle agitation (3 min) in HBSS containing 0.05% trypsin, 20 mM HEPES, 3 mM EGTA, 15 mM glucose and 2.5% BSA. INS–1 and dissociated islet cells were transduced with adenovirus carrying the fire-fly luciferase gene from Photinus Piralis (Ad-CMV-Luciferase) by rocking at a MOI of 100 for 4 hrs at room temperature. Cells were plated in 96 well plates. Dissociated islet cells were plated as a 1:1 suspension with matrigel (BD Biosciences, CA). Experiments were performed 48 hrs after viral transduction.

Luminescence measurements were performed in an Infinite M1000 plate reader (Tecan, Austria) controlled by either Megellan–6 or i-control 1.5 programs with readings taken at approximately 45 s intervals. After recordings in the plate reader were finished, light emitted by the cells in the wells was directly measured in a LAS 4000 Luminescent Image Analyzer (Fujifilm Life Science, CT) and quantified by densitometric analysis with the program Quantity One (Bio-RAd Laboratories).

### Islet insulin secretion

Four groups, each of 40 collagenase-isolated islets, were perifused in parallel and at a flow rate of 0.5 ml/min with Krebs-Ringer, buffered with 0.5 mM NaHCO_3_ and 20 mM HEPES, supplemented with 0.5% FA-free BSA (KRBH), and heated at 37°C. The perifusion pattern was similar in all the experiments. After a pre-perifusion period of 45 minutes under basal conditions (in the absence of nutrients or at 5 mM glucose), the perifusion medium was switched to one containing the test substances and maintained for the next 30 minutes. Finally, the medium was changed back to pre-perifusion conditions where it was maintained for the last 25 minutes. 70 mM KCl depolarization was always accompanied by addition of 250 μM diazoxide. The perifusate was collected at 1 minute intervals during the last 60–70 minutes of perifusion and its insulin concentration radioimmunologically measured. Pig insulin was radio-iodinated with Na^125^I [9] and rat insulin was used as a standard in the radioimmunoassay of insulin. Insulin antiserum was kindly provided by Dr. M. Villanueva-Peñacarrillo from the Department of Metabolism, Nutrition & Hormones, Fundación Jiménez Díaz, Madrid, Spain.

### Islet amino acid measurement

Islet amino acids (Asp, Glu, Ser, Gln, His, Gly, Thr, Arg, Tau, Ala, Tyr, and GABA) were separated by reverse-phase HPLC after precolumn derivatization with o-phthalaldialdehyde [10] and quantified by fluorescence detection. Three groups, each of 30 islets, were pre-incubated for 1 hour at 37°C in 70 μl KRBH containing 1 mM Gln. After discarding the medium and washing the islets twice with saline (100 μl each time), they were further incubated (in 70 μl KRBH) for a second hour at variable experimental conditions. Ca^2+^-omission means that the extracellular [Ca^2+^] is 0 mM and that 100 μM EGTA is added to KRBH. KCl depolarization was always accompanied by addition of 250 μM diazoxide. The increment of extracellular KCl was routinely compensated with a reduction of NaCl osmolarity. The incubations were stopped by placing the islets on ice. Aliquots of the incubation medium were also saved for measurements of amino acid release. The residual incubation medium was aspirated and the islets were washed twice with PBS (100 μl each time) and their amino acids extracted with 30 μl of 10% (w/v) 5-sulfosalicylic acid. One group of islets was taken from each preparation for the fluorometric determination of DNA [11].

### Islet adenine nucleotides measurement

Total rat islet ATP content was measured with the luciferin-luciferase system. Three groups, each of 25 islets, were incubated at 37°C for 60 minutes in 25 μl of KRBH. The incubation was stopped on acetone chilled with dry ice and 20 μl of 1.35 M perchloric acid (PCA) was added. PCA was neutralised and precipitated with 15 μl of 0.1 M Tris + 2.8 M of KHCO_3_. Supernatant aliquots (10 μl) of samples and ATP (or ADP) standard solutions (0–10 μM) treated in the same way were then mixed with 100 μl of D-luciferin solution (0.1 mM) in a 96 well plate. The emitted light was measured in a microplate reader (Synergy–2, Biotek) after addition of 10 μl of luciferase (0.1 mg/ml). Fire-fly luciferase (0.1 mg/ml) and D-luciferin (0.1 mM) were dissolved in a buffer containing 50 mM HEPES, 10 mM MgCl_2_, and 0.1% (w/v) FA-free BSA, and adjusted to pH 7.6. For the study of ATP uptake, the incubation medium was aspirated and islets washed thrice with KRBH containing 5 mM glucose and 50 μM mefloquine: first, the islets were pipetted out of the tubes after addition of 100 μl washing solution; secondly, they were extensively washed in a Petri dish containing 5 ml washing solution; thirdly, the islets were washed again with 100 μl in a clean tube and finally frozen after being resuspended in 25 μl KRBH containing 5 mM glucose. ADP was converted to ATP by pyruvate kinase (10 IU/ml) in the presence of 1 mM phosphoenolpyruvate in the islet PCA extract after neutralisation (2-fold dilution) and in ADP standard solutions (0–10 μM); an aliquot (5 μl) was used to measure the ATP content by the luciferin-luciferase system. The amount of ADP was calculated subtracting the ATP measured initially before the conversion. ATP release could not be detected with sufficient accuracy from incubated islets even in the presence of some specific inhibitors of the hydrolytic activity (100 and 200 μM sodium polytungstate and 100 μM 6N, N-diethyl-D-β,γ-dibromoethylene ATP, trisodium salt) because it was rapidly hydrolysed by ectonucleotidases (results not shown).

### Statistical analysis

Pairs of means ± S.E.M. were compared by Students’ t tests. Linear correlation between two variables were studied by regression analysis of the correlation coefficient and slope of the calculated straight line.

## Results

### Luciferin permeability of dispersed rat islet cells or INS–1 cells

**Fig 1:**
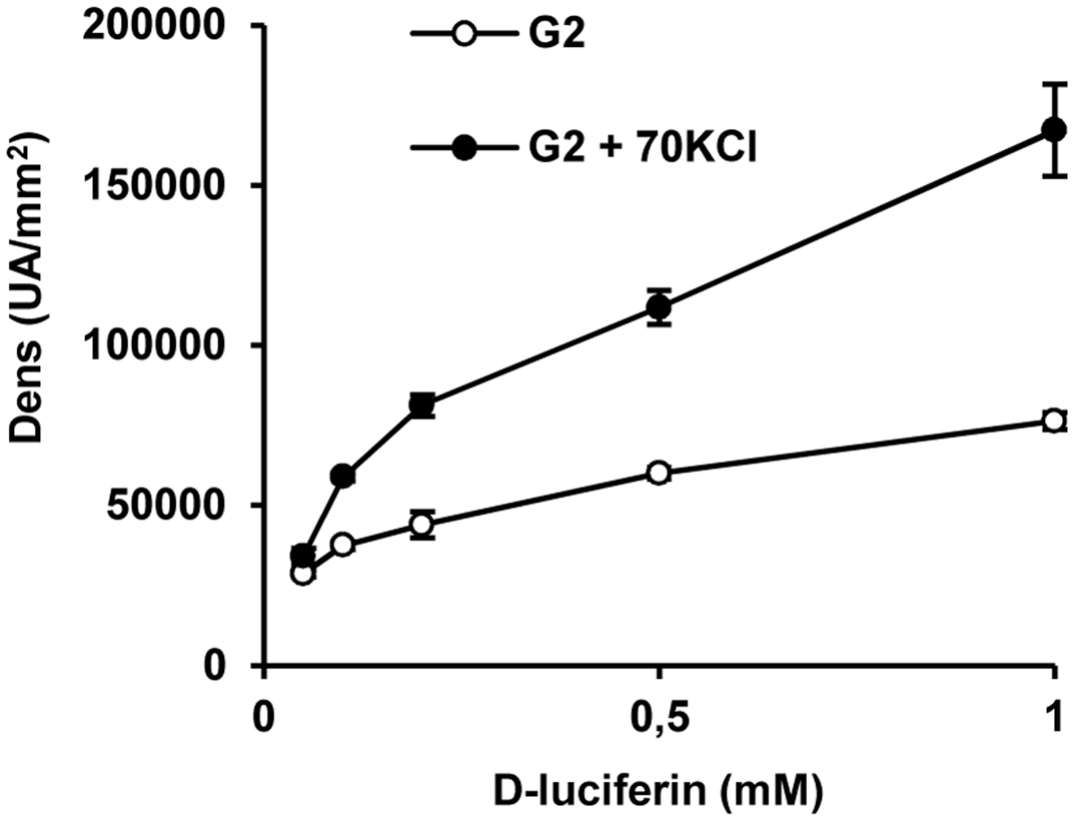
Dose-response relationship of light emission by INS–1 cells expressing the fire-fly luciferase gene with the medium D-luciferin concentration. INS–1 cells transduced with an adenovirus carrying the gene of fire-fly luciferase from Photinus Piralis were incubated at 2 mM glucose and varying concentrations of luciferin under control (4.7 mM KCl) or depolarizing conditions (70 mM KCl).

**Fig 2:**
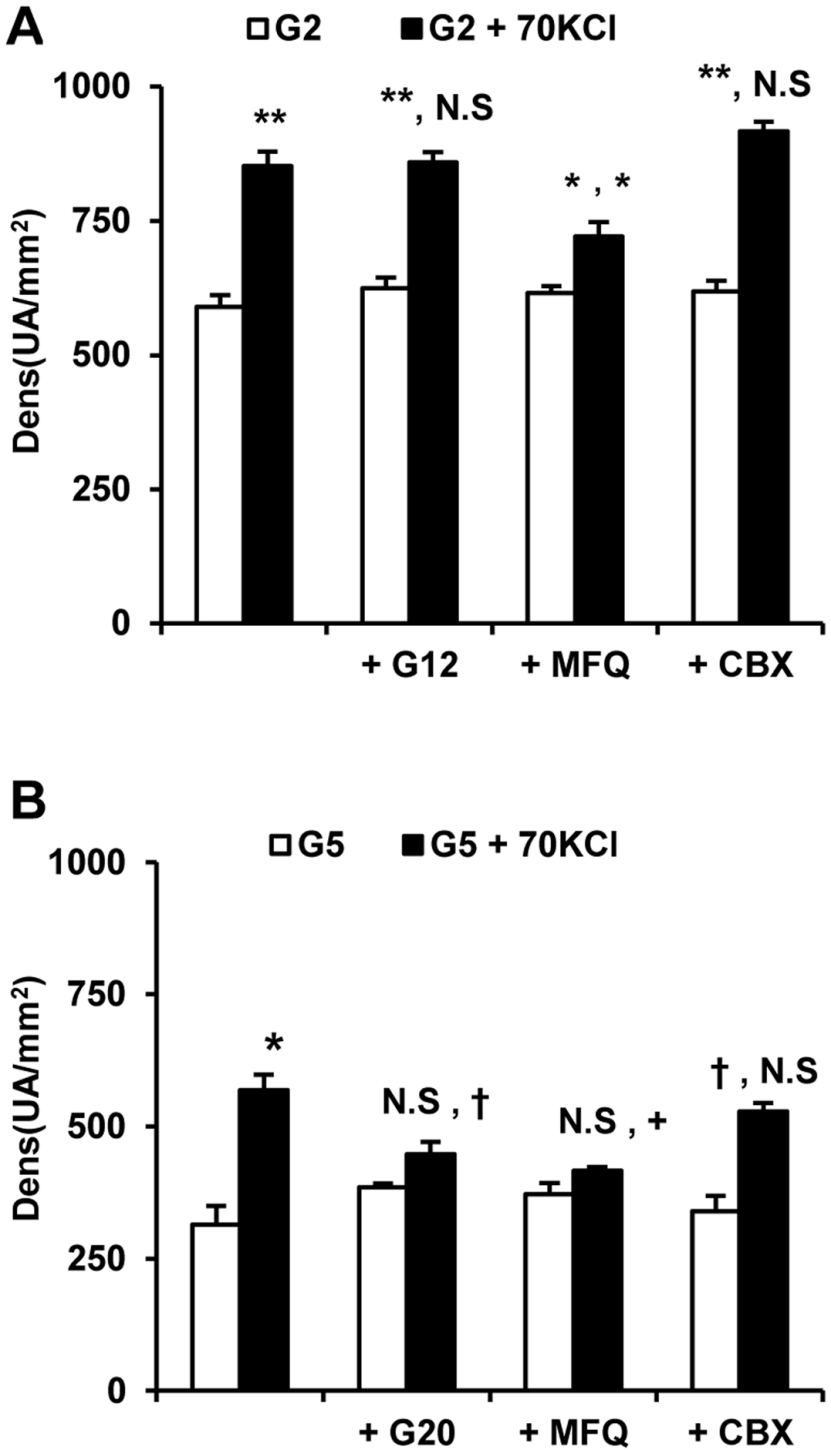
Effect of glucose and connexin inhibitors (mefloquine and carbenoxolone) on light emission induced by KCl-depolarization in INS–1 (A) and rat islet dispersed cells (B) expressing the fire-fly luciferase gene. INS–1 cells and dispersed rat islet cells, transduced with an adenovirus carrying the fire-fly luciferase gene, were incubated at control (4.7 mM KCl) or depolarizing (70 mM KCl) conditions in the presence of 1 mM D-luciferin. The effects of high glucose (G12, 12 mM and G20, 20 mM glucose), 50 μM mefloquine (MFQ), and 100 μM carbenoxolone (CBX) on light emission were investigated. (†p<0.05, +p<0.01, *p<0.005, and **p<0.001, compared with the white bar to the left in the first position and with the corresponding bar at 2 mM glucose (A) or 5 mM glucose (B) in the second position).

In order to explore changes in plasma membrane permeability induced by 70 mM KCl-depolarization dispersed islet cells or INS–1 cells were transduced with an adenovirus carrying the fire-fly luciferase gene. Luciferin was added to the incubation medium at 5 mM glucose and light production followed under control conditions (4.7 mM KCl) and after depolarization with 70 mM KCl. As shown in Fig 1, the amount of emitted light by transduced INS–1 cells augmented “exponentially” with the luciferin concentration (0.1 to 1.0 mM) in the incubation medium after depolarization with 70 mM KCl at 5 mM glucose. By contrast, non-depolarized control cells showed a flat response (Fig 1). In order to optimize the sensitivity, the highest luciferin concentration (1.0 mM) was systematically used in subsequent experiments. In these cells, light emission was not decreased by 20 mM glucose but it was diminished by 50 μM mefloquine (a specific connexin–36 inhibitor); 250 μM carbenoxolone (more specific pannexin inhibitor) had no effect (Fig 2). Transduced rat islet cells also showed an increased light emission after depolarization with 70 mM KCl in the presence of 1.0 mM luciferin (Fig 2). Light emission was significantly mitigated by 20 mM glucose and 50 μM mefloquine but not carbenoxolone.

### Islet exchange of amino acids

**Fig 3:**
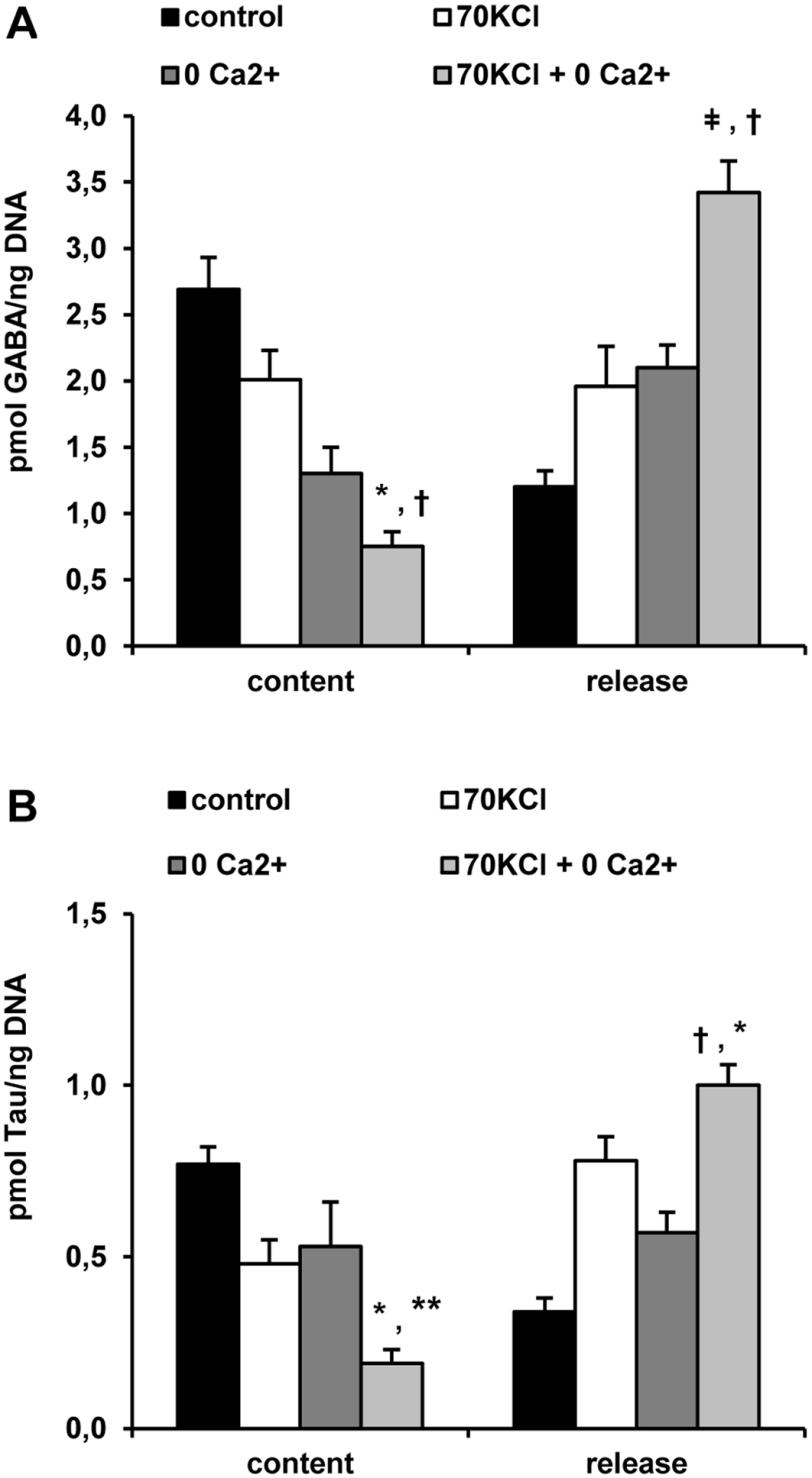
Effect of plasma membrane depolarization with 70 mM KCl (70 KCl) and/or extracellular Ca^2+^-omission on the islet content and release of GABA (A) and taurine (Tau) (B). Ca^2+^-omission (0 mM CaCl_2_ + 100 μM EGTA = 0 Ca^2+^) and 70 mM KCl depolarization (70 KCl + 250 μM diazoxide = 70 KCl) were tested on content and release of GABA and Tau in incubated rat islets. Three groups each of 30 rat islets were pre-incubated with 10 mM Gln and 5 mM glucose for 1 hour. After washing three times with 100 μl PBS to withdraw extracellular Gln, islets were further incubated for 1 hour at 37°C (in 70 μl of KRBH) with 5 mM glucose alone (control), in the absence of Ca^2+^ alone (0 Ca^2+^), in the presence of 70 mM (70 KCl) or under both Ca^2+^-omission and KCl-depolarization (0 Ca^2+^ + 70 KCl). At the end of incubation, an aliquot (50 μl) of incubation medium was taken off and the islets washed again three times with cold PBS (100 μl) to wash out remaining extracellular amino acids. Islets were finally extracted with 30 μl 10% (w/v) sulfosalycilic acid and stored frozen. Amino acids in the incubation media and islets extracts were separated by HPLC after derivatization with o-phtalaldialdehyde and quantified by fluorescence detection. (*p<0.0001, **p<0.005, ^†^p<0.02, ^ǂ^p<0.002 and ^#^p<0.01, compared with the corresponding mean value obtained with 70 mM KCl when placed in the first position or with the mean obtained with Ca^2+^-omission when placed in the second position).

**Fig 4:**
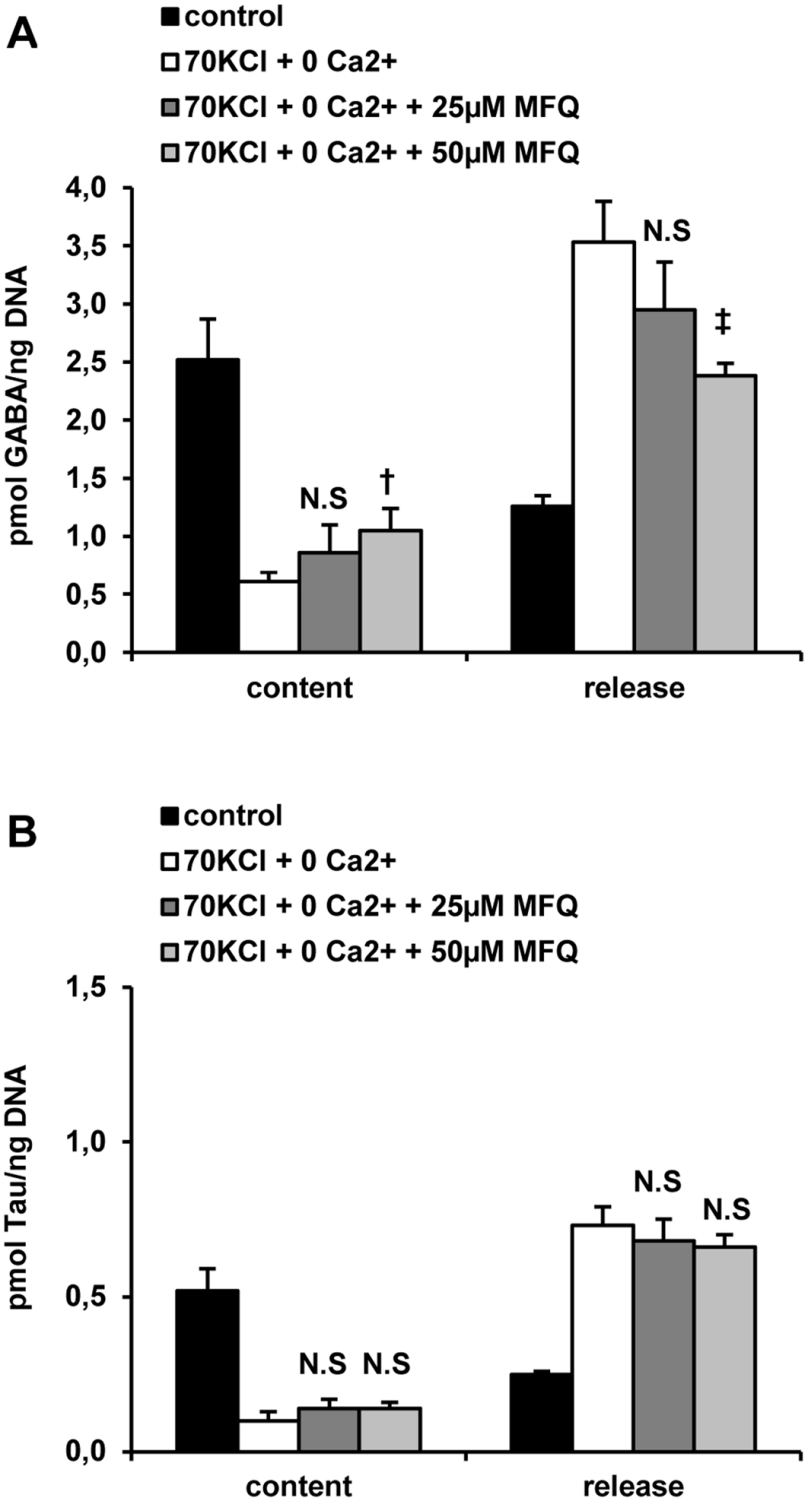
Effect of mefloquine (MFQ, a *Cx36* inhibitor) on the islet content and release of GABA (A) and taurine (Tau) (B) induced by plasma membrane depolarization with 70 mM KCl (70 KCl) and extracellular Ca^2+^-omission. Ca^2+^-omission (0 mM CaCl_2_ + 100 μM EGTA = 0 Ca^2+^) and 70 mM KCl depolarization (70 KCl + 250 μM diazoxide = 70 KCl) were tested on content and release of GABA and Tau in incubated rat islets. Three groups each of 30 rat islets were pre-incubated with 10 mM Gln and 5 mM glucose for 1 hour. After washing three times with 100 μl PBS to withdraw extracellular Gln, islets were further incubated for 1 hour at 37°C (in 70 μl of KRBH) with 5 mM glucose alone (control) or under Ca^2+^-omission and KCl-depolarization (0 Ca^2+^ + 70 KCl) in the absence or presence of 25 and 50 μM mefloquine. At the end of incubation, an aliquot (50 μl) of incubation medium was taken off and the islets washed again three times with cold PBS (100 μl) to wash out remaining extracellular amino acids. Islets were finally extracted with 30 μl 10% (w/v) sulfosalycilic acid and stored frozen. Amino acids in the incubation media and islets extracts were separated by HPLC after derivatization with o-phtalaldialdehyde and quantified by fluorescence detection. (N.S., † p< 0.04, and ‡ p< 0.01 compared with the corresponding control in the absence of MFQ).

70 mM KCl depolarization and Ca^2+^-omission separately increased the release of GABA and taurine (Tau) and correspondingly depleted their islet content (Fig 3A and 3B). These separate effects were strongly potentiated when the two conditions were applied together. The content and release of Asp and Glu were not so consistently affected by 70 mM KCl depolarization and Ca^2+^-omission (results not shown). Mefloquine, a specific inhibitor of connexin36, partially blocked the release of GABA stimulated by 70 mM KCl depolarization and Ca^2+^-omission at 50 but not at 25 μM and correspondingly increased islet amine content (Fig 4A). These data indicate that exchange of some amino acids occur via connexin–36 hemichannels in response to depolarization and Ca^2+^ removal. In contrast, the drug had no effect on either islet Tau depletion or release induced by 70 mM KCl depolarization and Ca^2+^-omission.

### Islet exchange of ATP and ADP

**Fig 5:**
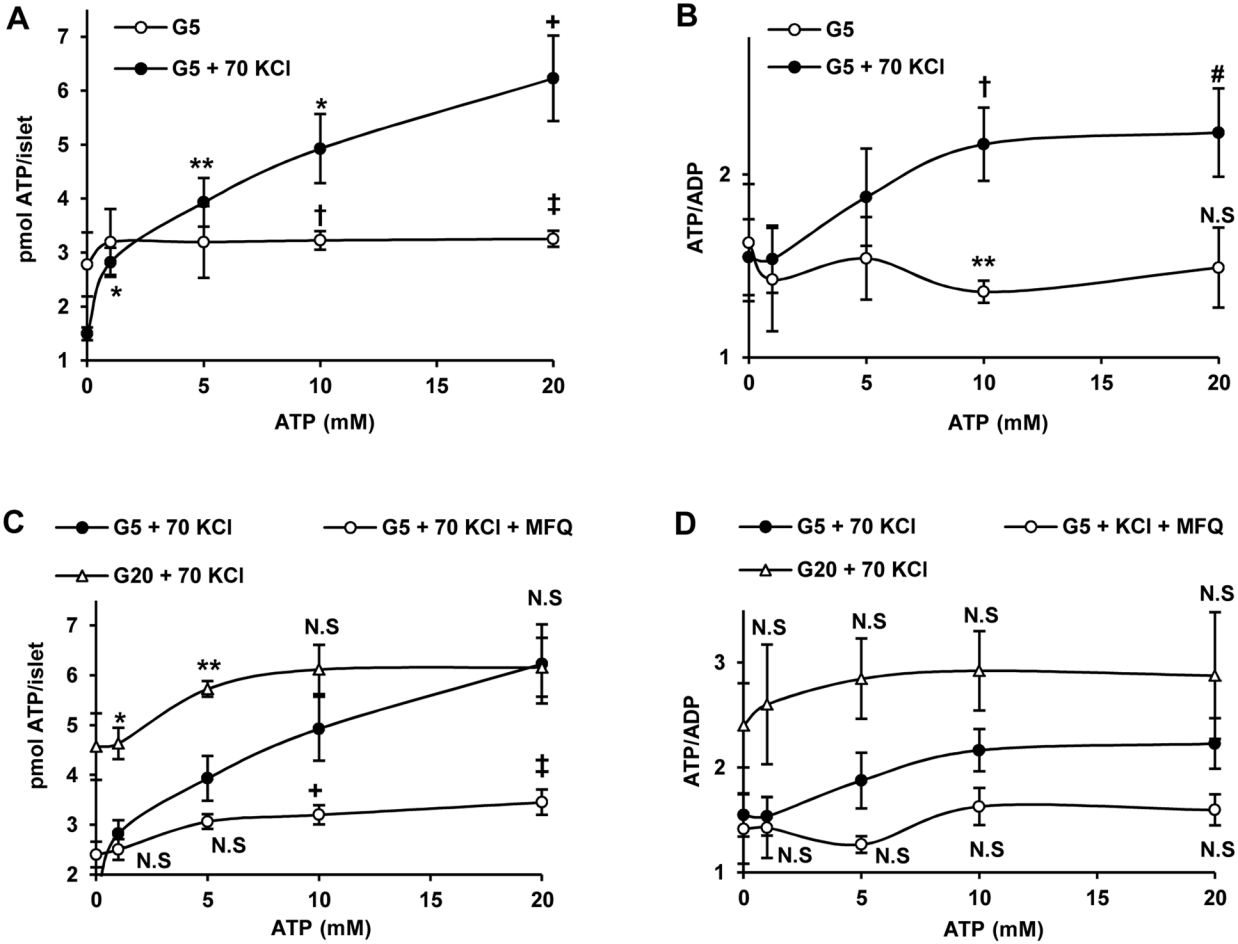
Dose-response curve of islet ATP content and ATP/ADP ratio with the extracellular nucleotide concentration under different conditions. Three groups of 25 islets each were incubated for 1 h at varying extracellular concentrations of ATP under the indicated experimental conditions and after extensive washing they were processed for the measurement of ATP and ADP contents, as described in detail in the Materials and methods section. A) Islets incubated at 5 mM glucose (G5) and physiological KCl concentration were compared with islets depolarized with 70 mM KCl (G5 +70KCl). ATP content (y) was significantly correlatedwith the extracellular nucleotide concentration (x) in KCl-depolarized islets (y = 0.23 x + 2.65; r^2^ = 0.68; n = 29; p<0.001, for both the correlation coefficient and the slope). B) The corresponding ATP/ADP values of previous experiments in A are shown. C) Effect of 20 mM glucose (G20) and 50μM mefloquine (MFQ) on ATP uptake by islets depolarized with 70 mM KCl (70KCl) at G5. D) Shows the corresponding ATP/ADP ratios of the experiments represented in C. (*p<0.01, **p<0.005, +p<0.001, #p<0.04, †p<0.03, and ‡p<0.02, NS = non-significant, compared with the corresponding non-depolarized control (open circles) in panels A and B or with the corresponding depolarized controls (closed circles) in panels C and D).

We have previously confirmed that KCl depolarized mouse islets lose ATP at 5 mM glucose and that the loss could be reversed in the presence of extracellular ATP [3]. We have investigated in more detail, using a wide concentration range, the kinetics of ATP uptake in rat islets (Fig 5). At 5 mM glucose, there was no significant uptake in the whole concentration range confirming that the washing procedure was efficient for removing the extracellular contaminating nucleotide (Fig 5A). 70 mM KCl depolarization induced a decreased ATP content at 5 mM glucose that was fully recovered by 1.0 mM extracellular ATP and further increased over the values of non-depolarized control islets at higher nucleotide concentrations (Fig 5A). A significant linear correlation was demonstrated between the ATP content and the extracellular nucleotide concentration in depolarized islets (legend of Fig 5A). The calculated islet ATP content at 1 mM extracellular ATP is 2.9 that is very close to the experimental nucleotide content in non-depolarized islets at 5mM glucose in the absence of extracellular ATP (2.7 ± 0.3 pmol/islet, as shown in Fig 5A). ATP uptake caused an increase of the islet ATP/ADP ratio at extracellular nucleotide concentrations above 5 mM (Fig 5B). Mefloquine (50 μM) blocked not only depolarization induced ATP loss at 5 mM glucose but also the uptake of extracellular ATP (Fig 5C). At 20 mM glucose, there was a slight but non-significant increase of ATP uptake at the different extracellular nucleotide concentrations and no decrease of islet ATP content in its absence (Fig 5C). Islet ATP/ADP ratio was increased by 20 mM glucose independently of the extracellular ATP concentration and reached similar values to those recorded in KCl depolarized islets incubated at 5 mM glucose and extracellular ATP (10 and 20 mM) (Fig 5D). Tolbutamide (0.5 mM) showed a trend to decrease both ATP and ADP (2.5 ± 0.1, n = 6, vs. 2.95 ± 0.3, n = 5, N.S. and 1.9 ± 0.1, n = 3 vs. 2.4 ± 0.3 pmol/islet, n = 3; N.S., respectively) in islets incubated at 5 mM glucose without modifying the ATP/ADP ratio (1.3 ± 0.07, n = 6, vs. 1.3 ± 0.04, n = 3; N.S.).

**Fig 6:**
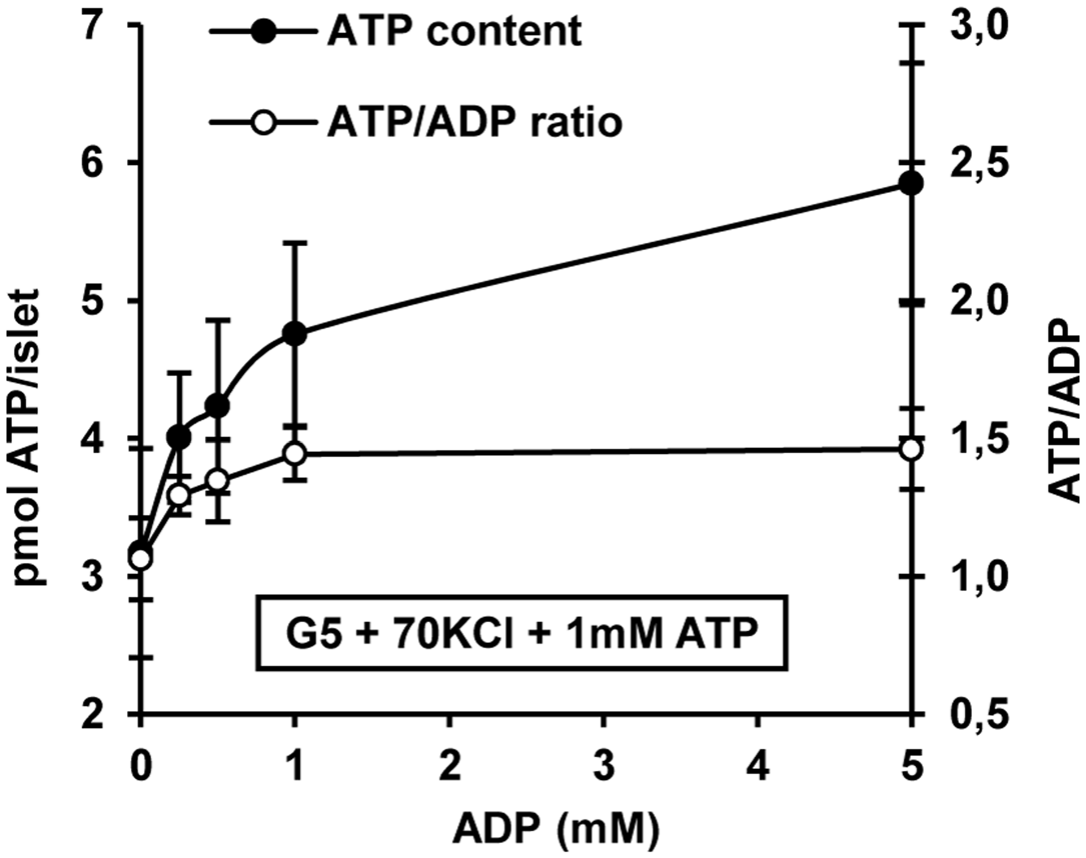
Dose-response curve of islet ATP content and ATP/ADP ratio with the extracellular ADP concentration under depolarizing conditions (70 mM KCl) and in the presence of a constant extracellular concentration of 1 mM ATP and 5 mM glucose. Three groups of 25 islets each were incubated for 1 h at varying extracellular concentrations of ADP under the indicated experimental conditions and after extensive washing they were processed for the measurement of ATP and ADP contents, as described in detail in the Materials and methods section.

ADP uptake was studied in islets depolarized with 70 mM KCl and incubated with 5 mM glucose plus 1.0 mM ATP (shown above to recover islet ATP from the depolarizing effect). Increasing ADP concentrations (0, 0.25, 0.5, 1.0 and 5.0 mM) increased islet ATP content without changing the intracellular ATP/ADP ratio (Fig 6). A positive but non-significant correlation was obtained between these two parameters (Fig 6). Neither was the islet ATP/ADP ratio significantly increased by extracellular ADP (Fig 6).

These data indicate that connexin36 channels allow nucleotide equilibration across the plasma membrane when they are opened by depolarization with KCl. The null point determined in these titrations, where ATP neither leaves nor enters, is 1 mM and is consistent with a cytosolic free ATP concentration of 1 mM as determined by others [12,13].

### ATP stimulation of insulin secretion in rat perifused islets

**Fig 7:**
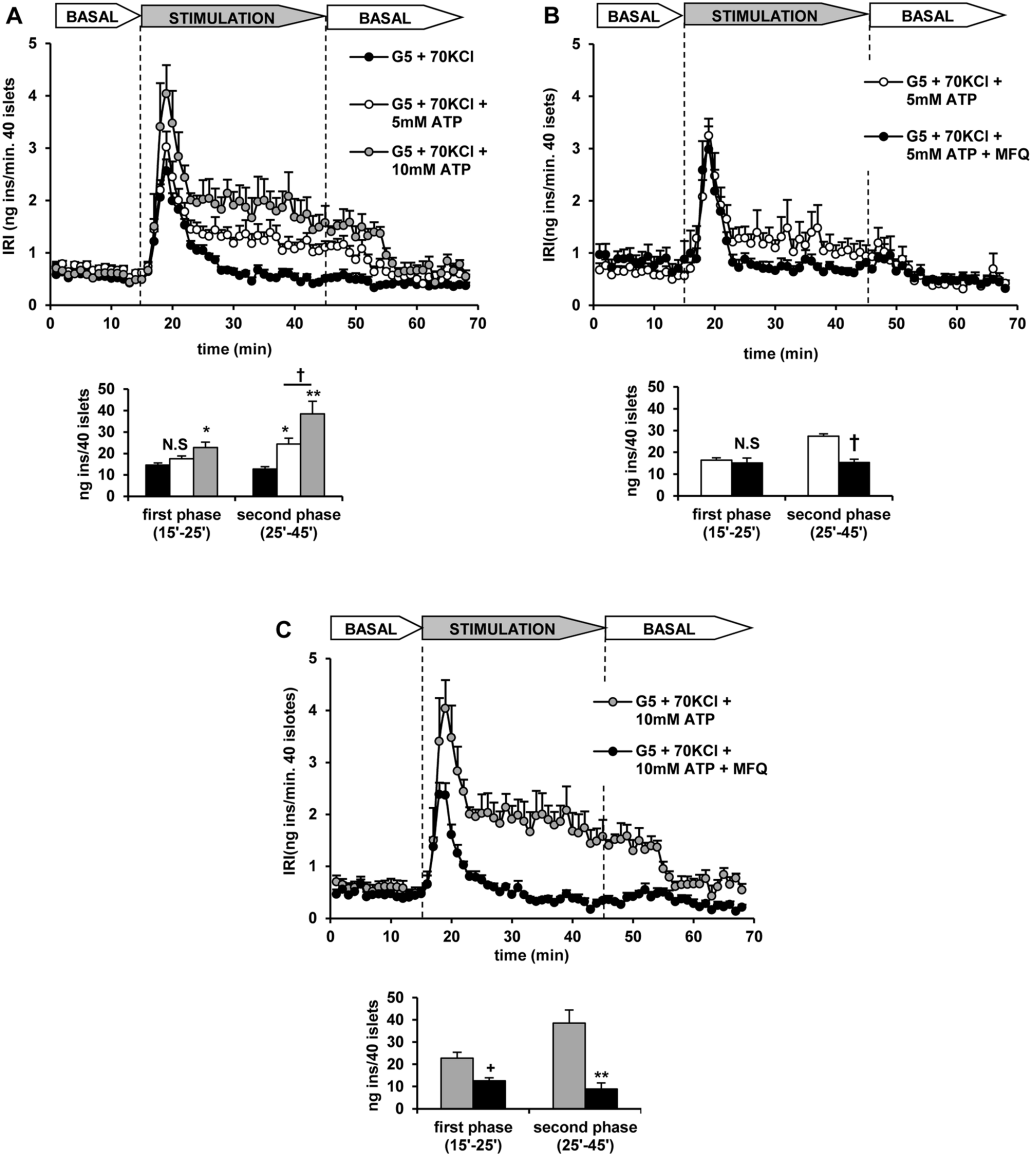
Potentiation of the monophasic secretory response of rat perifused islets to 70 mM KCl by extracellular ATP and its suppression by mefloquine. Groups of 40 islets each, pre-perifused with 5 mM glucose (G5) for 45 min, were stimulated for 30 min (between vertical broken lines) with 70 mM KCl at G5 in the absence or presence of extracellular ATP and 50μM mefloquine (MFQ). Pre-perifusion conditions were then re-established during the last 25 min. (A) KCl-depolarized islets were stimulated with 0, 5 or 10 mM ATP (black, white and grey symbols and bars, respectively). The secretory response to 5 mM (white symbols and bars) (B) or 10 mM ATP (grey symbols and bars) (C) were suppressed by mefloquine (black symbols and bars in both B and C panels). Symbols and bars represent means ± S.E.M. of 5 or 6 experiments (*p<0.005, **p<0.0001, †p<0.01, and +p<0.001, compared with their corresponding control: black bars in A, white and grey bars in B and D, respectively).

**Fig 8:**
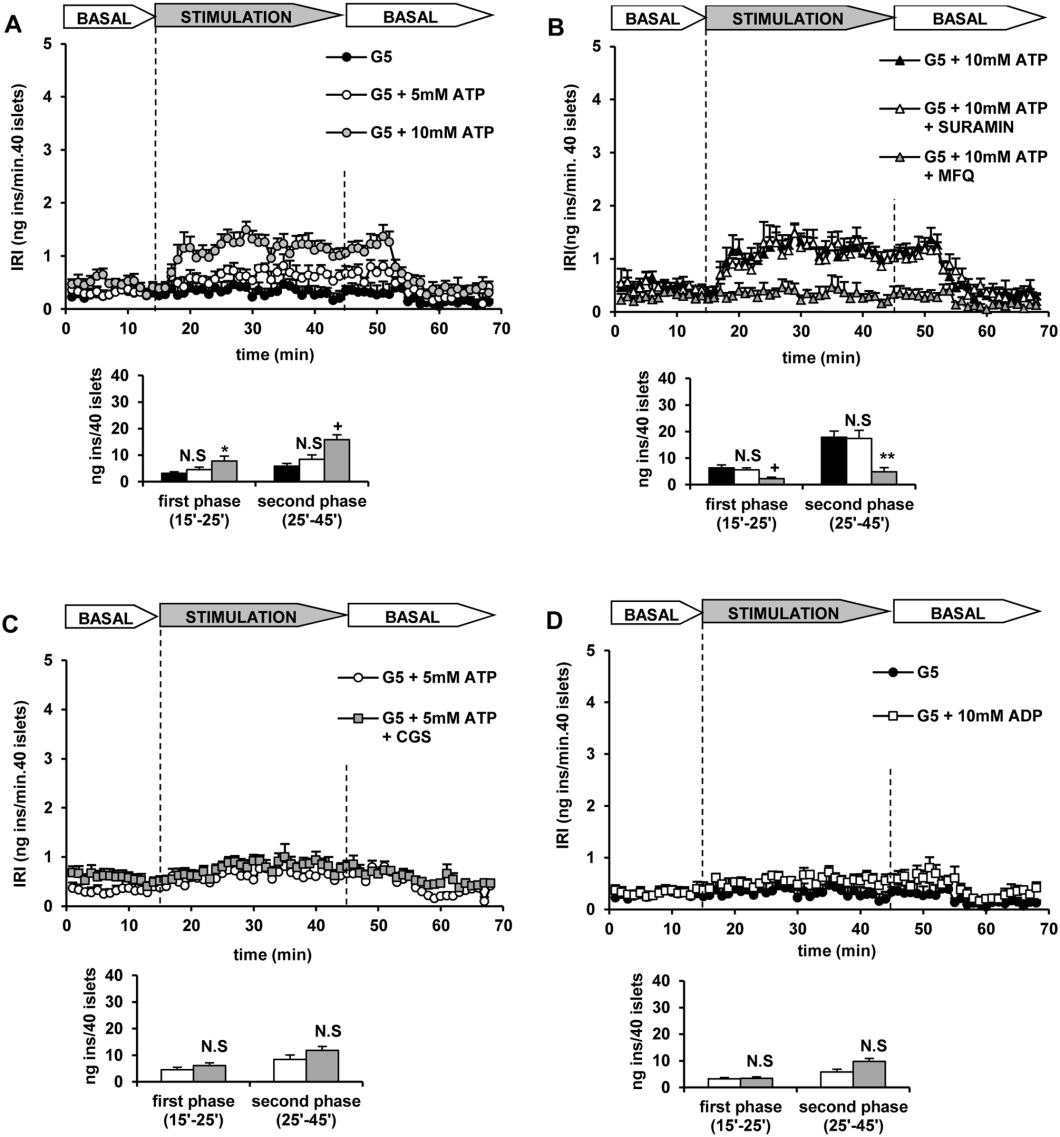
Stimulation of insulin secretion in rat perifused islets by extracellular ATP and ADP under a physiological KCl concentration (4.7 mM) and the effects of purinergic receptors’ antagonists and mefloquine. Groups of 40 islets each, pre-perifused with 5 mM glucose (G5) for 45 min, were stimulated for 30 min (between vertical broken lines) with a nucleotide at G5. Pre-perifusion conditions were then re-established during the last 25 min. A) Islets were stimulated by 5 (white symbols and bars) and 10 mM (grey symbols and bars) extracellular ATP. B) The stimulation by 10 mM ATP (black symbols and bars) was not affected by 50 μM suramin (purinergic receptor antagonist) (white symbols and bars) but strongly reduced by 50 μM mefloquine (MFQ) (grey symbols and bars). C) 5 mM ATP did not stimulate insulin secretion at G5 (white symbols and bars), neither was secretion potentiated by 50 μM CGS 15943, an adenosine receptor antagonist (grey symbols and bars). D) 10 mM ADP did not stimulate secretion at G5 (white and grey symbols and bars, respectively). Symbols and bars represent means ± S.E.M. of 5 to 6 experiments. (*p< 0.05, **p< 0.001 and +p< 0.01 compared with the corresponding control, black bars in A and B and white bars in C and D).

The influence of extracellular concentrations of ATP that increased its intracellular content and the ATP/ADP ratio were assayed on insulin secretion by KCl-depolarized islets. Islet depolarization with 70 mM KCl at 5 mM glucose induced a transient first phase of insulin secretion that returned to the basal values after 10 minutes (Fig 7A). Increasing the extracellular ATP concentration to 5 mM did not modify KCl-induced first phase but stimulated a sustained second phase of secretion (Fig 7A). 10 mM ATP increased both the first phase of secretion due to KCl depolarization and stimulated a second phase of higher magnitude than the one recorded with 5 mM ATP (Fig 7A). Control (non-depolarized) islets perifused at 5 mM glucose did not respond to 5 mM ATP (8.2 ± 1.7 n = 5, vs. 13.6 ± 2.7 ng insulin/40 islets x 30 min., n = 5; N.S.) (Fig 8A). However, 10 mM ATP triggered a small but sustained secretory response at 5 mM glucose that was 3-fold higher than the basal rate of secretion (8.2 ± 1.7, n = 5, vs. 25.4 ± 3.8 ng insulin/40 islets x 30 min., n = 5; p<0.001) (Fig 8A). It was not modified by 50 μM suramin, a known P2-purinergic receptor antagonist (24.1 ± 3.3, n = 6 vs. 22.9 ± 3.7 ng insulin/40 islets x 30 min., n = 6; N.S.) but strongly suppressed by 50μM mefloquine (Fig 8B). Neither was insulin secretion at 5 mM glucose and 5 mM ATP enhanced by 50 μM CGS 15943, a potent adenosine receptor antagonist (14.4 ± 2.2, n = 5 vs. 11.3 ± 1.4 ng insulin/40 islets x 30 min., n = 6; N.S.) (Fig 8C). In contrast to ATP, 10 mM ADP did not stimulate any secretory response in control, non-depolarized islets (13.1 ± 1.3, n = 6 vs. 8.2 ± 1.7 ng insulin/40 islets x 30 min., n = 5; N.S.) (Fig 8D).

**Fig 9:**
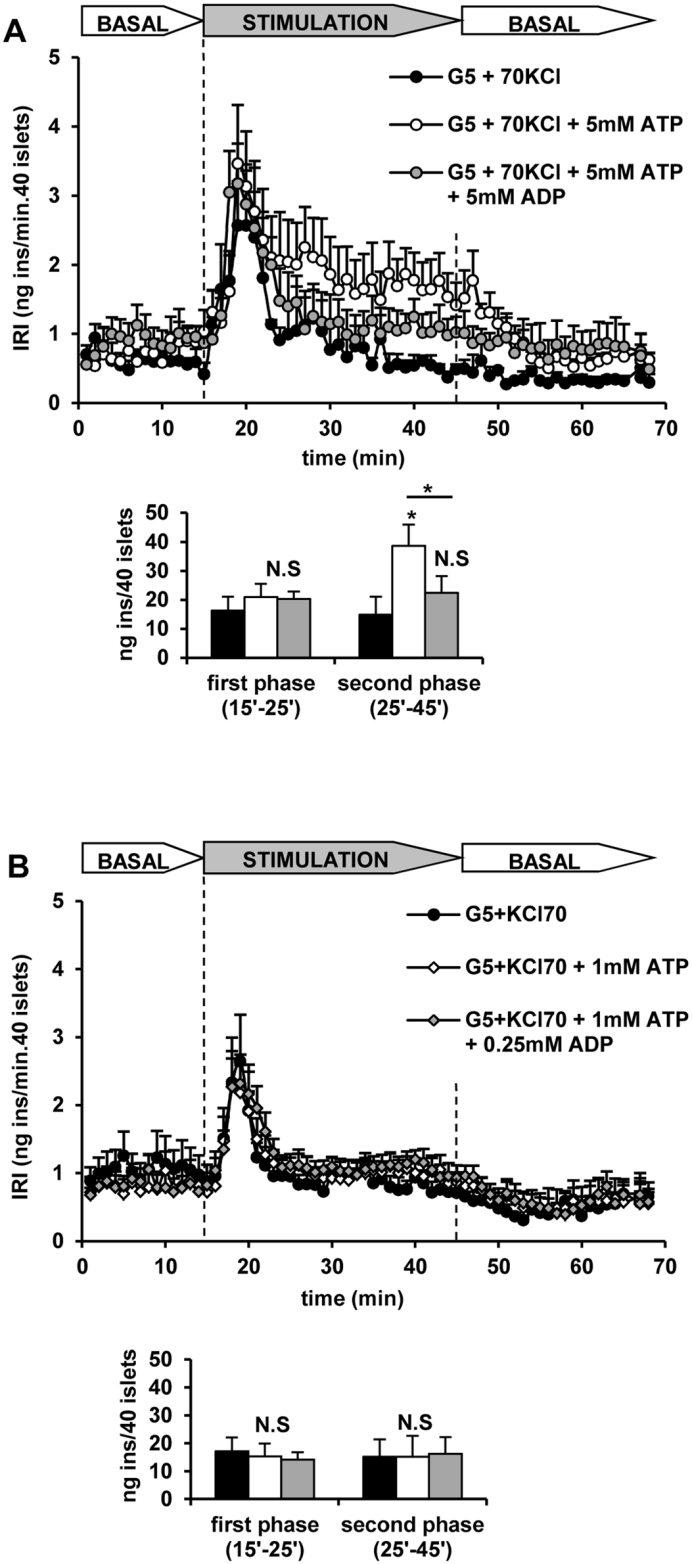
The secretory response of KCl-depolarized rat perifused islets to extracellular ATP depends on the extracellular ATP/ADP ratio. Groups of 40 islets each, pre-perifused with 5 mM glucose (G5) for 45 min, were stimulated for 30 min (between vertical broken lines) with 70 mM KCl (70 KCl) and ATP ± ADP at G5. Pre-perifusion conditions were then re-established during the last 25 min. A) Islets stimulated without (black symbols and bars) or with 5 mM ATP (with or without 5 mM ADP; grey and white symbols and bars, respectively). B) Similarly, islets were stimulated without (black symbols and bars) or with 1 mM ATP (with or without 0.25 mM ADP: grey and white symbols and bars, respectively). Symbols and bars represent means ± S.E.M. of 5 or 6 experiments. (*p<0.05, compared with the corresponding control in the absence of ATP).

It was shown above that islet cells’ plasma membrane depolarized with 70 mM KCl becomes permeable not only to ATP but also to ADP. We hypothesized that 5 mM ATP stimulation of insulin secretion in depolarized islets should be decreased by the simultaneous addition of 5 mM ADP if the intracellular ATP/ADP ratio is determined by the extracellular one. The results in Fig 9A show that the second phase of insulin release stimulated by 5 mM ATP was indeed decreased by more than 40% by 5 mM ADP. Addition of 1 mM ATP did not modify the secretory monophasic pattern induced by KCl depolarization (Fig 9B) consistent with a lack of effect on intracellular ATP. At this extracellular concentration, raising the extracellular ATP/ADP ratio to 4 (1 mM ATP + 0.25 mM ADP) did not stimulate a second phase of insulin secretion after the first phase induced by KCl (Fig 9B).

These findings support an effect of the adenine nucleotides on insulin secretion in addition to their well-documented ability to modulate the K_ATP_ channel.

### Effect of mefloquine on nutrient secretagogues’ stimulation of insulin secretion

Mefloquine (50 μM) was shown before (reference 3) to block insulin secretion and membrane exchange of ATP in KCl-depolarized mouse islets and currents stimulated by membrane depolarization in *Xenopus Laevis* oocytes overexpressing murine Cx36 cDNA (reference 3). It was tested on ATP-induced secretion to confirm that the stimulation is due to nucleotide diffusion through connexin 36 hemichannels opened by KCl depolarization. The drug, applied simultaneously with ATP completely blocked the potentiation by 10 mM ATP of the first phase of secretion triggered by 70 mM KCl depolarization (Fig 7C). It also strongly suppressed the stimulation of a second phase of secretion by either 5 or 10 mM ATP in depolarized islets (Fig 7B and 7C). Moreover, mefloquine also completely blocked the stimulation of insulin secretion by 10 mM ATP in control, non-depolarized islets (Fig 8B).

**Fig 10:**
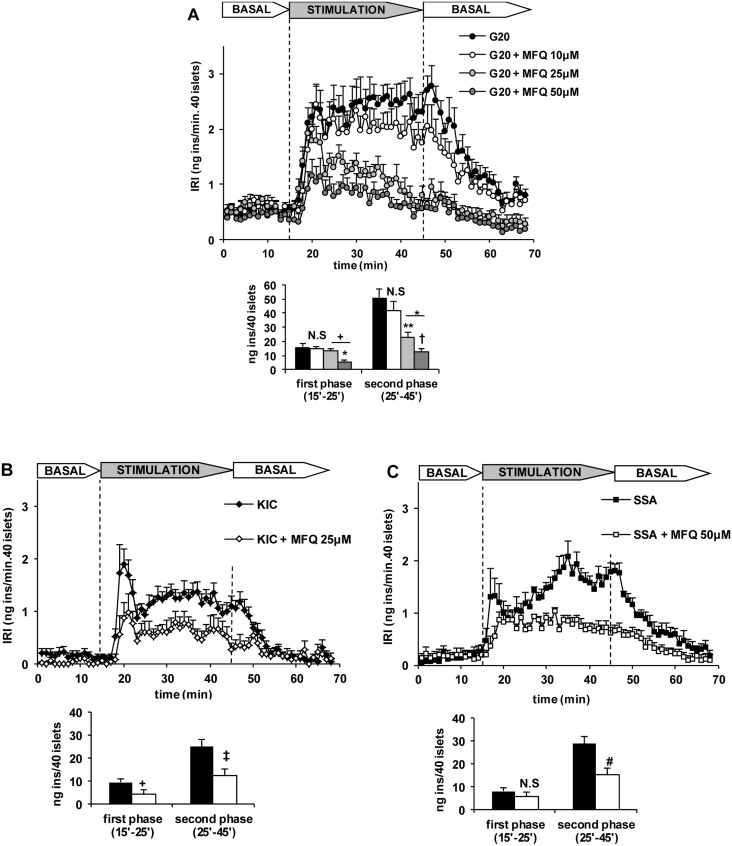
Effect of mefloquine (MFQ) on the stimulation of insulin secretion by 20 mM glucose (G20), 10 mM KIC and 10 mM semialdehyde succinic acid (SSA) in rat perifused islets under physiological conditions. Groups of 40 islets each, pre-perifused for 45 min, were stimulated for 30 min (between vertical broken lines) with different nutrient secretagogues. Pre-perifusion conditions were then re-established during the last 25 min. A) Islets pre-perifused with 5 mM glucose were then stimulated with 20 mM glucose (G20) (black symbols and bars) and varying MFQ concentrations (10, 25 and 50 μM: white, light grey and intense grey symbols and bars, respectively). B) Islets pre-perifused in the absence of any substrate were then stimulated with 10 mM KIC without (black symbols and bars) or with 25 μM MFQ (white symbols and bars). C) Islets pre-perifused without any susbstrate were stimulated with 10 mM SSA without (black symbols and bars) or with 50 μM MFQ (white symbols and bars). Symbols and bars represent means ± S.E.M. of 5–6 experiments. (*p<0.005, **p<0.001, †p<0.0005, +p<0.05, ‡p<0.01, and #p<0.003, compared with the corresponding control (black bars).

In our hands, 50 μM mefloquine behaved as a specific inhibitor of connexin 36 in β-cells and it was therefore expected to close not only hemichannels but also gap junction channels. Genetic deletion of connexin36 interrupts the synchronization of the intracellular calcium responses among individual β-cells within the islets and deranges the stimulation of insulin secretion [14–15]. We checked whether connexin36 inhibition with mefloquine interfered with a normal stimulation of insulin secretion by nutrient secretagogues. The drug exerted a dose-dependent decrease of the insulin secretory response to 20 mM glucose: at 10 μM it had no effect but at higher concentrations (25 and 50 μM) it progressively diminished both the first and second phase of secretion (Fig 10A). At 25 μM, it also suppressed the biphasic response of perifused islets to 10 mM KIC in the absence of glucose (Fig 10B). Similarly, the biphasic insulin release induced by semialdehyde succinic acid was strongly decreased by 50 μM mefloquine (Fig 10C).

## Discussion

INS–1 cells and dispersed islet cells expressing fire-fly luciferase emitted a low background of visible light under control conditions (physiological KCl concentration) when luciferin was added to the incubation medium. However, the light intensity increased exponentially with the medium luciferin concentration when the cells were depolarized with 70 mM KCl. We conclude that depolarization increases the plasma membrane permeability of INS–1 and islet cells allowing the entrance of luciferin and the activity of endogenously expressed luciferase on intracellular ATP. The suppressing effect of mefloquine on light emission under depolarizing conditions strengthens the idea that the permeability increase of the plasma membrane is due to the opening of Cx36 hemichannels by the increased KCl concentration.

Both adenine nucleotides and amino acids are released from islets depolarized with 70 mM KCl and their release is also suppressed by mefloquine supporting the participation of open hemichannels in this process. As calcium omission seems to potentiate islet metabolite release, it cannot be attributed to an increased exocytosis stimulated by KCl-depolarization. Many connexin isoforms are able to form not only intercellular, gap junction, channels but open solitary hemichannels for rapid exchange of ions, second messengers, cofactors, and metabolites between the cell interior and the interstitial space [6, 7, 8]. Connexon hemichannels are known to be opened by membrane depolarization and divalent cation omission besides other mechanisms [7]. The effects of mefloquine on islet cells has been exhaustively studied and discussed before [3] and its action seems to be rather specific in the range 10 to 50 μM. Stronger support for the participation of Cx36 hemichannels in the increased permeability of β-cells at least to extracellular adenine nucleotides was obtained in islets from transgenic mice with a germinal knockout of the Cx36 gene: Cx36^-/-^ islets did not exchange ATP with the incubation medium and mefloquine suppressed this exchange only in Cx36^+/+^ and Cx36^+/-^ but had no effect in Cx36^-/-^ islets (3). A direct inhibitory effect of mefloquine on Cx36 could also be demonstrated in *Xenopus Laevis* oocytes where it suppressed Cx36-hemichannels’ mediated currents [3].

Depolarization induced opening of Cx36 hemichannels makes β-cells’plasma membrane permeable to small molecules with a mass below 1KD but their specificity for diffusible solutes has not yet been fully characterized. We checked whether KCl-depolarized islet cells could take up extracellular ATP. It was demonstrated that islet ATP content increased in a wide range (1 to 20 mM) of extracelular ATP concentrations under depolarizing conditions. This concentration-dependent ATP-uptake was almost completely suppressed by 50 μM mefloquine and did not happen in islets incubated at a physiological KCl concentration. At 1 mM, extracellular ATP restored the islet adenine nucleotide content of depolarized islets to the physiological levels of control, non-depolarized islets. Assuming that extracellular ATP equilibrates with the intracellular pool within the time-course of the experiment (1 h), one may conclude that the physiological intracellular concentration at 5 mM glucose is close to 1 mM. This value coincides with the resting ATP concentration in the cytosol, the mitochondrial matrix and the submembrane space found in primary rat islet β-cells expressing fire-fly luciferase in different compartments [12]. A similar value for the submembrane ATP concentration has been found in mammalian cells using a mutant Kir6.2 K_ATP_ channel subunit sensitive to the nucleotide in the range 0.1 to 10 mM [13]. As the islet ATP content increased with its extracellular concentration, the ATP/ADP ratio also increased to values characteristic of glucose-stimulated islets. By contrast, increasing extracellular ADP in the presence of 1 mM ATP did not increase the nucleotide ratio in depolarized islets at 5 mM glucose. This seems to suggest that in this case active metabolism is needed to increase the phosphorylation potential.

Extracellular ATP at 5 and 10 mM stimulated a second phase of insulin secretion in KCl-depolarized islets in a concentration dependent manner that was associated with similar increases of ATP content and ATP/ADP ratio above the values found in non-depolarized islets. Whereas ATP stimulated a second phase of release at 5 mM, the stimulation by 10 mM was stronger and it also potentiated the first phase induced by KCl depolarization. That means that an increase of cytosolic ATP may also contribute not only to the amplifying but to the triggering pathway responsible for the development of the second and first phase of stimulation of insulin release by glucose, respectively [16]. An increase of intracellular ATP is not exclusively inhibiting K_ATP_-channels but it also probably exerts an additional role amplifying insulin granule exocytosis during both phases of insulin secretion [17, 18].

Amplification of insulin secretion by extracellular ATP in depolarized islets seems to depend on its absolute concentration and on the ATP/ADP ratio. At 1 mM extracellular ATP the recorded ATP/ADP ratio was close to 1 and no secretion stimulation took place; clamping the extracellular ATP/ADP ratio to 4 (1 mM ATP + 0.25 mM ADP) did not allow the stimulation of any second phase release. It might be concluded that at the resting intracellular ATP concentration no stimulation of insulin secretion occurs even though the ATP/ADP ratio is raised above the stimulatory values recorded at 20 mM glucose. By contrast, the second phase of release triggered by 5 mM extracellular ATP is strongly reduced in the presence of 5 mM ADP (extracellular ATP/ADP = 1). These results are analogous to the suppression by 5 mM ADP of the capacitance increase due to 3 mM ATP (ATP/ADP ratio = 0.6) at high [Ca^2+^]_i_ in patch-clamped mouse β-cells [18].

In control, non-depolarized islets, incubated at 5 mM glucose, insulin secretion was slightly stimulated by 10 but not 5 mM extracellular ATP. This small secretory response was not antagonized by suramin (P2-purinergic antagonist) [19] but was almost completely suppressed by mefloquine. These results support the interpretation that the secretory effect of extracellular ATP at a physiological KCl concentration is mainly due to its uptake through Cx36 hemichannels that seem to be partially open at resting, hyperpolarized, conditions. Failure of CGS 15943, an adenosine receptor antagonist, speaks against the participation of an inhibitory tone on insulin secretion triggered by ATP degradation.

In summary, islets depolarized with KCl exhibited an increased plasma membrane permeability to adenine nucleotides whose loss makes β-cells less competent for secretion, as demonstrated by the failure of KIC to stimulate a second phase of secretion [1]. Pre-exposure of islets to a depolarizing KCl concentration at 5 mM glucose also provokes a partial blockade of the subsequent insulin response to 20 mM glucose [2]. Therefore, KCl induced loss of ATP and perhaps other metabolites should be taken in consideration at the time of interpreting the results obtained under KCl depolarizing conditions. KCl-permeabilized islets did not lose insulin content within the time frame of the experiments and responded, reversibly and in a connexin36-sensitive manner, to increases of extracellular ATP in the expected way. They may constitute an adequate experimental model to explore the messenger role of ATP and other metabolites in insulin secretion.
